# Polymorphisms in *CACNA1A*, *CACNA1C*, and *CACNA1H* Genes in Korean Pediatric Patients with Developmental Delay and Intellectual Disability: A Focus on Epilepsy Comorbidity

**DOI:** 10.3390/genes16070767

**Published:** 2025-06-29

**Authors:** Ji Yoon Han

**Affiliations:** 1 Department of Pediatrics, College of Medicine, The Catholic University of Korea, Seoul 06591, Republic of Korea; han024@catholic.ac.kr; Tel.: +82-42-220-9540; Fax: +82-42-221-2925; 2 Department of Pediatrics, Daejeon St. Mary’s Hospital, College of Medicine, The Catholic University of Korea, Daejeon 34943, Republic of Korea

**Keywords:** *CACNA1A*, *CACNA1C*, *CACNA1H*, polymorphism, developmental delay, intellectual disability, epilepsy

## Abstract

**Background:** Developmental delay and intellectual disability (DD/ID) are frequently accompanied by epilepsy, and growing evidence implicates variants in voltage-gated calcium channel genes in their pathogenesis. This study aimed to investigate the association of polymorphisms in *CACNA1A*, *CACNA1C*, and *CACNA1H* with DD/ID and epilepsy comorbidity in Korean children. **Methods:** We retrospectively analyzed 141 pediatric patients diagnosed with DD/ID who underwent whole-exome sequencing (WES) and were not found to have pathogenic monogenic variants. Nine single-nucleotide polymorphisms (SNPs) across *CACNA1A*, *CACNA1C*, and *CACNA1H* were selected based on functional annotation scores and prior literature. Genotype data were extracted from WES variant files, and allele and genotype frequencies were compared with control data from the gnomAD East Asian population and the Korean Reference Genome Database (KRGDB). Subgroup analyses were performed according to epilepsy comorbidity. **Results:** The *CACNA1A* rs16023 variant showed a significantly higher B allele frequency in DD/ID patients than in both control datasets and was also associated with epilepsy comorbidity. Genotype distribution analysis revealed that the BB genotype of rs16023 was more frequent in patients with epilepsy. **Conclusions:** The *CACNA1A* rs16023 variant may contribute to genetic susceptibility to DD/ID and epilepsy in Korean children, potentially through regulatory mechanisms. These findings support the relevance of calcium channel genes in neurodevelopmental disorders and highlight the importance of integrating functional annotation in variant prioritization.

## 1. Introduction

Developmental delay and intellectual disability (DD/ID) affect approximately 1–3% of the pediatric population and are frequently accompanied by additional neurological comorbidities, most notably epilepsy [1]. Increasing evidence indicates that both monogenic mutations and polygenic influences contribute to the pathogenesis of DD/ID and its associated clinical manifestations [2]. Among the gene families implicated in neurodevelopmental disorders, voltage-gated calcium channel genes—including *CACNA1A* (encoding Cav2.1), *CACNA1C* (Cav1.2), and *CACNA1H* (Cav3.2)—are of particular interest due to their central roles in neuronal signaling, excitability, and neurodevelopment [3]. These genes encode subunits of P/Q-type, L-type, and T-type calcium channels, respectively, and have been associated with a broad range of neurodevelopmental and neuropsychiatric conditions [4,5]. Beyond the nervous system, mutations in voltage-gated calcium channel genes have been implicated in a wide range of systemic disorders. For instance, *CACNA1C* variants associated with Timothy syndrome are known to cause cardiac arrhythmias due to delayed repolarization [6]. T-type channel genes such as *CACNA1H* have also been linked to primary aldosteronism and renal hypertension [7], while calcium-channel-mediated signaling plays a role in immune cell activation [8]. These findings highlight the importance of comprehensive pediatric evaluation when interpreting variants in calcium channel genes. Pathogenic variants in these genes have been linked to epilepsy, autism spectrum disorder, and ataxia, while common single-nucleotide polymorphisms (SNPs) may modulate susceptibility or influence phenotypic severity [3,9,10,11,12]. However, few studies have examined the association of these polymorphisms with DD/ID in pediatric populations, particularly in the context of epilepsy comorbidity.

Although whole-exome sequencing has significantly improved the diagnostic yield in children with DD/ID, a substantial proportion of cases remain without a clearly identifiable pathogenic variant [2]. In such instances, common polymorphisms—especially those located in regulatory or non-coding regions—may contribute to disease risk by subtly affecting gene expression or neuronal function [13]. Recent studies support a polygenic model in which both rare deleterious mutations and common variants interact to influence the phenotypic spectrum of neurodevelopmental disorders [14]. Therefore, functional SNPs in genes involved in neuronal signaling may help explain genetic risk in patients lacking monogenic diagnoses.

Among such gene families, voltage-gated calcium channel genes continue to receive particular attention for their roles in synaptic signaling and brain development [15]. For example, *CACNA1A* mutations have been classically associated with episodic ataxia type 2, familial hemiplegic migraine, and spinocerebellar ataxia, as well as epileptic encephalopathies and developmental syndromes [16]. *CACNA1C* has been identified as a susceptibility locus in multiple psychiatric disorders, including bipolar disorder, schizophrenia, and autism spectrum disorder [17]. Similarly, *CACNA1H* variants have been linked to various epilepsy subtypes, particularly childhood absence epilepsy [18]. Despite the growing body of literature on these genes, data remain limited regarding their contribution to neurodevelopmental phenotypes in East Asian populations.

This study aims to address this gap by investigating the frequency of selected SNPs in *CACNA1A*, *CACNA1C*, and *CACNA1H* in Korean children with DD/ID, and by evaluating their association with epilepsy comorbidity. Although the term neurodevelopmental disorders (NDDs) encompasses a wide range of conditions, including autism spectrum disorder (ASD), attention-deficit/hyperactivity disorder (ADHD), ID, and language disorders, this study specifically focuses on DD and/or ID, which represent key components within the broader NDD spectrum.

## 2. Materials and Methods

### 2.1. Participants

A total of 254 pediatric patients diagnosed with DD/ID underwent chromosomal microarray (CMA) and/or whole-exome sequencing (WES) as part of clinical evaluation. Among them, 113 were diagnosed with defined genetic syndromes, including chromosomal abnormalities, pathogenic CNVs, or monogenic variants. The remaining 141 patients without a definitive genetic diagnosis were selected for the present study focusing on calcium channel gene polymorphisms. This subset was specifically selected to evaluate the contribution of common polymorphisms in genetically undiagnosed cases. Among them, 87 (61.7%) had comorbid epilepsy. Participants were aged between 1 month and 18 years and visited the Department of Pediatric Neurology at Daejeon St. Mary’s Hospital between 1 January 2019 and 31 December 2024. This study was approved by the Institutional Review Board of Daejeon St. Mary’s Hospital (IRB No. DC25RASI0024, approval date: 31 May 2025), and written informed consent was obtained from all parents or legal guardians.

Developmental delay and intellectual disability (DD/ID) were defined according to age-appropriate diagnostic criteria. For children under 5 years of age, developmental delay (DD) was diagnosed when there was a significant delay (≥2 standard deviations below the mean) in one or more developmental domains (gross motor, fine motor, speech/language, cognitive, or social) as measured by standardized tools such as the Korean Developmental Screening Test (K-DST) or the Bayley Scales of Infant and Toddler Development, Third Edition (Bayley-III). For children aged 5 years and older, intellectual disability (ID) was diagnosed according to the DSM-5 criteria, which require an IQ score below 70 using age-appropriate instruments such as the Wechsler Preschool and Primary Scale of Intelligence (WPPSI-IV); the Wechsler Intelligence Scale for Children, Fourth Edition (WISC-IV); or the Kaufman Assessment Battery for Children, Second Edition (KABC-II), along with concurrent adaptive dysfunction assessed using the Vineland Adaptive Behavior Scales, Second Edition (VABS-II). Patients were included in this study if they met criteria for either DD (if <5 years) or ID (if ≥5 years), and had undergone whole-exome sequencing without a definitive monogenic diagnosis. The severity of DD/ID was classified based on DQ or IQ scores as follows: mild (50–69), moderate (35–49), and severe/profound (<35), in accordance with DSM-5-based clinical practice. Motor delay was defined as a significant delay (≥2 standard deviations below the mean) in gross or fine motor development appropriate for age. Language delay was defined as a delay of ≥2 standard deviations in receptive and/or expressive language skills, based on standardized developmental assessments such as the K-DST or Bayley-III. Sleep disturbance was defined as the presence of persistent difficulties in initiating or maintaining sleep, frequent nighttime awakenings, or irregular sleep–wake patterns, based on parent-reported history obtained during clinical interviews. Epilepsy was defined according to the 2014 ILAE criteria, including either ≥2 unprovoked seizures more than 24 h apart or one unprovoked seizure with high recurrence risk. Patients in the non-epilepsy group were followed clinically for at least one year after enrollment, and none showed evidence of seizure activity during this period.

Exclusion criteria included non-genetic causes of DD/ID, such as:

- Documented perinatal brain injury (e.g., hypoxic-ischemic encephalopathy, grade III/IV intraventricular hemorrhage);
- Acquired structural brain abnormalities on MRI (e.g., periventricular leukomalacia, diffuse cortical atrophy);
- History of central nervous system infections (e.g., meningitis, encephalitis);
- Known metabolic disorders or traumatic brain injuries with sequelae;
- Diagnosed chromosomal syndromes or aneuploidies, unless separately analyzed.

### 2.2. SNP Selection and Genotype Extraction

Nine tagging SNPs in the CACNA1A, CACNA1C, and CACNA1H genes were selected based on the following criteria:

- Minor allele frequency (MAF) ≥ 0.01 in East Asian populations, as our aim was to analyze common or low-frequency polymorphisms rather than rare or ultra-rare variants;
- Location in functionally important regions (coding exons, 3′ UTRs, enhancers, splice junctions);
- High predicted impact: Combined Annotation Dependent Depletion (CADD) score > 10 or RegulomeDB score ≤ 3a;
- Illumina Final SNP Score ≥ 0.6, indicating genotyping confidence.

Genotype data for these selected SNPs were extracted from WES variant call format (VCF) annotation files using chromosomal positions aligned to the GRCh37/hg19 human genome reference. GRCh37 was selected due to its compatibility with our institutional annotation pipeline and its alignment with public databases such as gnomAD v2.1.1 and KRGDB, which are also based on GRCh37. The SNPs included *CACNA1A* (rs16023, rs7249246, rs2270655), *CACNA1C* (rs1006737, rs4765905, rs2007044), and *CACNA1H* (rs2753326, rs2753325, rs2235631). A summary of their genomic locations, MAFs in East Asians, CADD and RegulomeDB scores, and predicted functional relevance is provided in Table 1.

## 3. Results

A total of 141 pediatric patients with DD/ID were included in the analysis, of whom 45 (31.9%) had comorbid epilepsy (Table 2). The mean age of the epilepsy group was significantly higher than that of the non-epilepsy group (10.89 ± 5.26 vs. 5.70 ± 3.92 years, *p* < 0.001). The mean age at epilepsy diagnosis was 5.50 ± 5.12 years. There was no significant difference in sex distribution between the groups (*p* = 0.868). With respect to the severity of DD/ID, the proportions of mild, moderate, and severe/profound cases did not significantly differ between the groups (*p* = 0.253, 0.134, and 0.925, respectively). Motor delay was observed in 15% of the total cohort, with no significant intergroup difference (*p* = 0.263). However, language delay was significantly more frequent in the epilepsy group (82%) than in the non-epilepsy group (56%) (*p* = 0.003). Autism spectrum disorders were present in 30% of all patients, with comparable frequencies between the epilepsy and non-epilepsy groups (*p* = 0.760). Sleep disturbances were relatively uncommon (11% overall) and did not differ significantly between groups (*p* = 0.866). Regarding family history, 16% of patients had a family history of DD/ID, and this did not differ between groups (*p* = 1.000). A family history of epilepsy was significantly more common in the epilepsy group (18%) compared to the non-epilepsy group (2%) (*p* = 0.002).

A total of nine SNPs in the *CACNA1A*, *CACNA1C*, and *CACNA1H* genes were selected based on their reported associations with neurodevelopmental disorders and epilepsy. The basic characteristics and functional annotations of these variants are summarized in Table 2. The CADD scores for these SNPs ranged from 8.7 to 15.7, indicating varying degrees of predicted deleteriousness. RegulomeDB scores suggested that several variants may have potential regulatory effects, particularly those with scores between 1f and 2b. A final score was calculated by integrating CADD and RegulomeDB results to prioritize variants with the highest predicted pathogenic relevance. rs16023 in *CACNA1A*, located in the 3′ UTR, showed high functional relevance (final score = 0.847), supported by a CADD score of 12.3 and a RegulomeDB score of 1f. rs7249246 (intron) and rs2270655 (missense) in *CACNA1A* also demonstrated elevated functional scores. SNPs in *CACNA1C* and *CACNA1H* were primarily intronic or synonymous, though several exhibited moderate-to-high CADD scores. These scores were used to complement allele frequency comparisons, allowing for a more biologically grounded variant prioritization. Variants with higher final scores were selected for further analysis in relation to epilepsy comorbidity.

**Figure 1:**
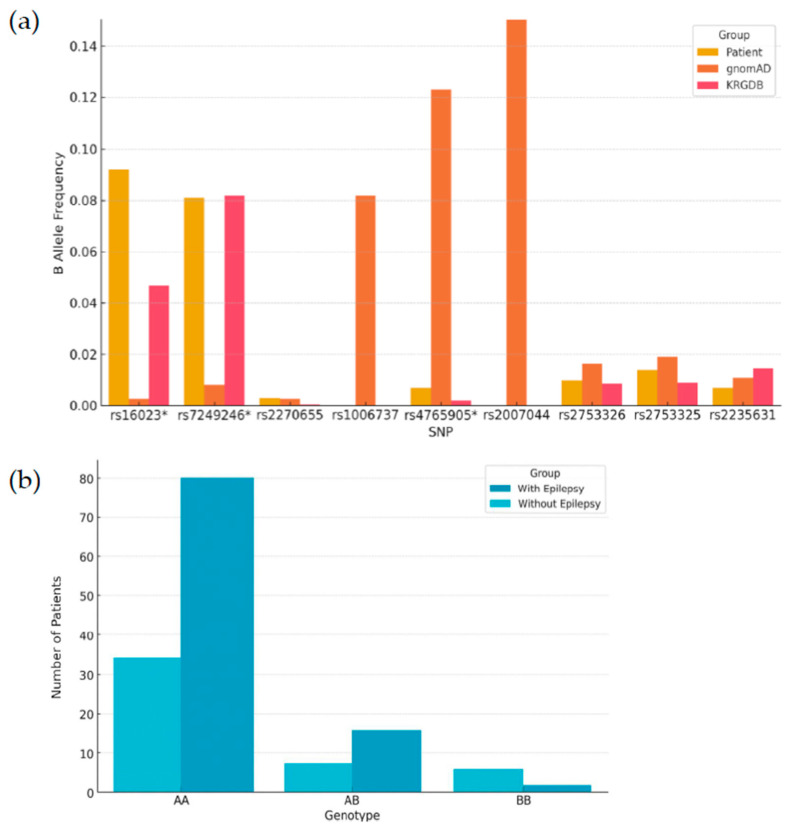
Allele frequency comparison between pediatric patients with DD/ID and the general population. (**a**) Comparison of B allele frequencies of nine selected SNPs (rs16023, rs7249246, rs2270655, rs1006737, rs4765905, rs2007044, rs2753326, rs2753325, rs2235631) among DD/ID patients, the gnomAD East Asian population, and the Korean Reference Genome Database. The asterisk (*) indicates statistical significance (*p* < 0.05) in allele frequency differences. (**b**) Genotype distribution of rs16023 (AA, AB, BB) in patients with developmental delay/intellectual disability, stratified by epilepsy comorbidity (with vs. without epilepsy). A significant difference in genotype distribution was observed (*p* < 0.05).

We analyzed the allele frequencies of nine single-nucleotide polymorphisms (SNPs) in the *CACNA1A*, *CACNA1C*, and *CACNA1H* genes among pediatric patients with DD/ID. Comparisons were performed using allele frequency data from the Genome Aggregation Database (gnomAD) East Asian population as the primary control group (Table 3), and from the Korean Reference Genome Database (KRGDB) as a secondary control group for ethnic-specific validation (Supplementary Table S1). Among the analyzed SNPs, rs16023 (*CACNA1A*) showed the most robust association with the patient group. The frequency of the B allele was significantly higher in patients (9.2%) compared to gnomAD controls (0.27%), yielding an odds ratio (OR) of 37.51 (95% CI: 21.59–65.19, *p* < 0.001). This result remained statistically significant when compared to the Korean population reference (KRGDB B freq = 0.0468, OR = 2.07, 95% CI: 1.37–3.13, *p* = 0.0016). Of the nine SNPs analyzed, only rs16023 showed a statistically significant difference in B allele frequency between the patient group and both control datasets (gnomAD and KRGDB). All other SNPs demonstrated significance in only one reference population or showed no significant difference in either, indicating variability depending on the control dataset used. Figure 1 illustrates the comparative B allele frequencies of all nine SNPs between the patient group and two population datasets (gnomAD East Asian and KRGDB). Notably, rs16023, rs7249246, and rs4765905 showed significantly different allele frequencies in DD/ID patients compared to gnomAD controls (*p*  <  0.05), suggesting potential relevance to disease susceptibility.

This table presents the genomic position, allele frequency in the East Asian population (MAF, based on gnomAD), and predicted functional impact of selected SNPs in the CACNA1A, CACNA1C, and CACNA1H genes. CADD scores indicate the deleteriousness of variants, with higher scores suggesting greater pathogenic potential [19]. RegulomeDB scores reflect regulatory function, where lower scores (e.g., 1a–2b) provide stronger evidence for regulatory activity [20]. Final scores integrate CADD and RegulomeDB data to estimate the overall functional relevance of each variant. SNPs were prioritized based on potential relevance to neurodevelopmental and epileptic phenotypes.

We analyzed the allele frequencies of nine single-nucleotide polymorphisms (SNPs) in the *CACNA1A*, *CACNA1C*, and *CACNA1H* genes among pediatric patients with developmental delay/intellectual disability (DD/ID). Comparisons were performed using allele frequency data from the gnomAD East Asian population as the primary control group (Table 3), and from the KRGDB as a secondary control group for ethnic-specific validation (Supplementary Table S1). Among the analyzed SNPs, rs16023 (*CACNA1A*) showed the most robust association with the patient group. The frequency of the B allele was significantly higher in patients (9.2%) compared to gnomAD controls (0.27%), yielding an odds ratio (OR) of 37.51 (95% CI: 21.59–65.19, *p* < 0.001). This result remained statistically significant when compared to the Korean population reference (KRGDB B freq = 0.0468, OR = 2.07, 95% CI: 1.37–3.13, *p* = 0.0016). Of the nine SNPs analyzed, only rs16023 showed a statistically significant difference in B allele frequency between the patient group and both control datasets (gnomAD and KRGDB). All other SNPs demonstrated significance in only one reference population or showed no significant difference in either, indicating variability depending on the control dataset used. Figure 1 illustrates the comparative B allele frequencies of all nine SNPs between the patient group and two population datasets (gnomAD East Asian and KRGDB). Notably, rs16023, rs7249246, and rs4765905 showed significantly different allele frequencies in DD/ID patients compared to gnomAD controls (*p*  <  0.05), suggesting potential relevance to disease susceptibility.

To investigate genotype-level differences according to epilepsy comorbidity in children with DD/ID, we compared the distribution of genotypes across nine SNPs between patients with epilepsy (*n* = 45) and those without epilepsy (*n* = 96) (Table 4). Among the nine SNPs analyzed, rs16023 (*CACNA1A*) demonstrated a statistically significant difference in genotype distribution between the two groups (*p* = 0.0215). The BB genotype was observed in 5 of 45 patients with epilepsy, compared to only 1 of 96 patients without epilepsy. For all other SNPs, including rs7249246, rs2753326, and rs2753325, no statistically significant differences in genotype distributions were found between the epilepsy and non-epilepsy groups (*p* > 0.05). Notably, for many SNPs such as rs2270655, rs1006737, and rs2007044, the BB genotype was absent or extremely rare in both groups, which may limit the power to detect meaningful differences. Among the SNPs analyzed, rs16023 (*CACNA1A*) demonstrated a statistically significant difference in genotype distribution between the two groups (*p* = 0.0215). As shown in Figure 1, the BB genotype of rs16023 was observed in 11.1% of epilepsy cases but only 1.0% of non-epilepsy cases, supporting its possible role in epilepsy susceptibility.

## 4. Discussion

In our cohort, patients with DD/ID and comorbid epilepsy exhibited distinct clinical characteristics compared to those without epilepsy, as summarized in Table 1. Notably, the epilepsy group was significantly older at the time of assessment and demonstrated a higher prevalence of language delay (82% vs. 56%, *p* = 0.003) and a family history of epilepsy (18% vs. 2%, *p* = 0.002). The higher prevalence of language delay observed in patients with epilepsy may be explained by the disruptive effects of recurrent seizures or interictal epileptiform discharges on neural circuits involved in language development, particularly during critical periods of early childhood. Temporal lobe epilepsy, which often involves language-related cortical areas, has also been associated with impaired language acquisition and abnormal language lateralization in pediatric populations [21]. These findings suggest that epilepsy-related neurophysiological disturbances may underlie the increased frequency of language delay in this group. While prior studies have linked calcium channel variants to various neurodevelopmental disorders such as ASD, ID, and ADHD, our study narrows the focus specifically to DD/ID, thereby providing a more targeted analysis of this core subgroup within the NDD spectrum. These findings suggest that epilepsy in children with DD/ID may be associated with more severe neurodevelopmental impairment and a stronger genetic predisposition. All patients in the non-epilepsy group were followed for at least 12 months without any seizure occurrence, minimizing the risk of misclassification due to age-dependent epilepsy onset. Nonetheless, future longitudinal studies will be needed to confirm these findings over time. This observation aligns with previous studies indicating that children with both DD/ID and epilepsy often exhibit greater cognitive and language deficits and are more likely to have familial or genetic etiologies compared to those without seizures [22,23]. While no significant differences were observed for other clinical features such as motor delay or autism spectrum disorder, the absence of statistical significance may reflect limited sample size or clinical heterogeneity within the DD/ID population. In this study, DD and ID were grouped under the term DD/ID to reflect the continuum of cognitive impairments observed in pediatric populations. However, we acknowledge that DD and ID may differ in their developmental timing, clinical trajectory, and potentially in their underlying genetic architecture. Similarly, comorbid conditions such as ASD, psychotic symptoms, and language disorders may represent biologically distinct subgroups with specific genetic susceptibilities. While subgroup analyses were beyond the scope of this study due to sample size limitations, future work incorporating deeper phenotypic stratification may uncover gene–phenotype correlations that are currently masked by diagnostic heterogeneity.

To evaluate the functional relevance of the selected SNPs, we integrated CADD and RegulomeDB scores. Among these, *CACNA1A* rs2270655 and rs16023 exhibited high final scores (0.874 and 0.847, respectively), suggesting strong pathogenic potential. Notably, rs2270655 is a missense variant previously associated with infantile spasms and epilepsy [24,25], while rs16023 is a regulatory variant located in the 3′ untranslated region (UTR), previously linked to developmental disorders and epilepsy [19,20]. Among the *CACNA1C* variants, rs1006737 and rs4765905 demonstrated high predicted regulatory activity, with RegulomeDB scores of 1f and 2b, respectively, and have been consistently implicated in neuropsychiatric disorders such as autism, schizophrenia, and epilepsy [26]. Similarly, rs2753326 in *CACNA1H*, which had the highest final score (0.917), has been associated with absence epilepsy and may function through enhancer-like regulatory mechanisms [27]. These integrated functional scores support the biological plausibility of observed genotype–phenotype associations in our cohort and highlight the value of prioritizing variants not solely based on statistical association, but also on predicted functional impact. Nonetheless, experimental validation through functional studies is warranted to confirm the pathogenic or regulatory roles of these variants.

Among the nine SNPs analyzed, *CACNA1A* rs16023 showed the most robust association with DD/ID. The B allele frequency was significantly higher in the patient group compared to both gnomAD East Asian (0.092 vs. 0.0027, *p* < 0.001) and KRGDB populations (0.092 vs. 0.0468, *p* = 0.0016), with corresponding odds ratios suggesting substantial enrichment. Furthermore, when stratified by epilepsy comorbidity, the rs16023 variant remained significantly associated with epilepsy status (*p* = 0.0215), with the BB genotype detected exclusively in the epilepsy group. These results suggest that rs16023 may contribute to both the susceptibility to DD/ID and the development of epilepsy in this population. Although rs16023 is located in a non-coding region (3′ UTR), its high CADD and RegulomeDB scores suggest potential post-transcriptional regulatory functions.

The *CACNA1A* gene encodes the α1A subunit of the *p*/Q-type voltage-gated calcium channel (Cav2.1), which plays an essential role in synaptic transmission, neuronal excitability, and cerebellar development [28]. This gene is highly expressed in the cerebral cortex, hippocampus, thalamus, and Purkinje cells—regions critically involved in neurodevelopment and epileptogenesis [29,30]. Pathogenic variants in *CACNA1A* have been implicated in a wide spectrum of neurological phenotypes, including episodic ataxia type 2, familial hemiplegic migraine, epileptic encephalopathy, and autism spectrum disorder [14,29,30]. The rs16023 variant, located in the 3′ UTR of *CACNA1A*, may affect mRNA stability, localization, or translational efficiency by altering microRNA (miRNA) binding sites [31,32]. Such post-transcriptional regulation could influence the expression levels of calcium channel subunits during critical periods of brain development. Prior studies have also suggested that CACNA1A 3′ UTR polymorphisms may modulate susceptibility to epilepsy and neurodevelopmental disorders [17,22]. The strong functional annotation scores for rs16023 (CADD = 12.3; RegulomeDB = 1f) support its potential regulatory role. Given its significant enrichment in the DD/ID cohort and specific association with epilepsy, rs16023 may serve as a candidate modifier or susceptibility locus. It is important to note that while the association between the rs16023 variant and DD/ID with epilepsy is statistically robust, this study does not establish a causal relationship. The observed correlation may be mediated by intermediate molecular mechanisms—such as altered mRNA regulation or network-level neuronal excitability—which remain to be elucidated. Experimental studies will be required to confirm the functional consequences and mechanistic relevance of this variant. Future studies using patient-derived neuronal models or cell-based assays are needed to investigate its mechanistic effects on gene regulation and disease pathogenesis.

Interestingly, the B allele frequency of rs16023 showed notable differences between the gnomAD East Asian (0.0027) and Korean KRGDB (0.0468) populations, indicating possible ethnic or population-specific genetic variation. These findings emphasize the necessity of using ancestry-matched control data in genetic association studies, as failure to account for population differences may lead to spurious or underestimated associations [33,34].

The CACNA1A rs16023 variant demonstrated an elevated frequency of the B allele in patients with developmental delay, with an even more pronounced association observed in those with comorbid epilepsy. Previous studies have further demonstrated that disruption of *CACNA1A* function may impair synaptic plasticity and contribute to abnormal neuronal network excitability, mechanisms that likely underlie both neurodevelopmental delay and seizure susceptibility [9,35,36]. Although rs16023 is a non-coding variant located in the 3′ untranslated region (UTR), its potential regulatory function cannot be excluded—particularly in light of its significant association with disease phenotypes in our cohort. Functional annotation scores, including a CADD score of 12.3 and a RegulomeDB score of 1f, further support its biological relevance. These findings underscore the importance of *CACNA1A* not only in normal neurodevelopment but also in the pathogenesis of epilepsy.

Further studies are warranted to validate the functional impact of rs16023 and to elucidate its regulatory role. Future research should incorporate experimental approaches such as in vitro reporter gene assays, electrophoretic mobility shift assays, transcriptomic profiling, or studies using patient-derived neuronal models to confirm the pathogenic significance of this SNP. This study focused specifically on three voltage-gated calcium channel genes—*CACNA1A*, *CACNA1C*, and *CACNA1H*—due to their well-established roles in neurodevelopmental disorders and epilepsy. These genes encode distinct channel types (P/Q-type, L-type, and T-type, respectively) and have been widely studied in both monogenic and polygenic disease contexts. However, other calcium channel genes such as *CACNA1B*, *CACNA1D*, and *CACNA1E* also play important roles in neuronal excitability and synaptic function. The exclusion of these genes represents a limitation of this study and may restrict the scope of our findings. Future studies encompassing a broader set of calcium channel genes may help further elucidate the genetic architecture of DD/ID and epilepsy. Despite the strengths of our study, several limitations must be acknowledged. Although the cohort size is relatively substantial for a rare pediatric neurodevelopmental condition, the sample size may still limit statistical power to detect modest effect sizes or rare variant interactions. An additional limitation of our study is the use of WES rather than whole-genome sequencing. While WES enables efficient detection of coding variants, it may miss noncoding regulatory variants—including those in distal enhancers, promoters, or intronic regions—that contribute to polygenic risk. Given the growing evidence that complex neurodevelopmental phenotypes are shaped by both coding and noncoding variation, future studies employing WGS may yield a more comprehensive understanding of the genetic architecture of DD/ID and epilepsy. Moreover, this study lacked experimental validation to confirm the functional consequences of the identified variants. It is also possible that environmental or epigenetic factors, which were not assessed, could have influenced the observed associations. These observations support the notion that regulatory and polygenic mechanisms may underlie the associations identified in our study. Integrative approaches combining genomic, transcriptomic, and clinical data will be essential to further elucidate the multifactorial etiology of neurodevelopmental disorders such as DD/ID with epilepsy. Future investigations incorporating multi-omic approaches—including transcriptomic, epigenomic, and electrophysiological studies—will be essential to elucidate the underlying mechanisms and to validate the causal roles of implicated variants.

## 5. Conclusions

This study identified a significant association between the *CACNA1A* rs16023 variant and both DD/ID and epilepsy comorbidity in Korean pediatric patients. The increased frequency of the B allele and its genotype-specific distribution support a potential role for rs16023 in conferring genetic susceptibility to neurodevelopmental disorders. These findings highlight the relevance of voltage-gated calcium channel genes in the pathogenesis of DD/ID and emphasize the importance of population-specific genetic analyses that incorporate functional annotation to guide variant prioritization. Given its regulatory location in the 3′ untranslated region and its predicted pathogenic potential, rs16023 may serve as a candidate biomarker for neurodevelopmental risk stratification in Korean children. Given the significant enrichment and predicted regulatory relevance of the rs16023 variant, it may serve as a potential biomarker for identifying children at elevated risk for DD/ID and epilepsy. With further validation, this variant could be incorporated into targeted gene panels or polygenic risk prediction models, including those used in prenatal or early-childhood screening. Such applications may support early diagnosis, surveillance, and potentially individualized interventions. Future research incorporating experimental validation—such as transcriptomic profiling, functional assays, or patient-derived neuronal models—will be essential to confirm the regulatory impact of rs16023 and to further elucidate its mechanistic role in disease pathogenesis.

## Figures and Tables

**Table 1:** Summary of selected SNPs and their functional impact in CACNA1A, CACNA1C, and CACNA1H.

Genes	SNP ID	Alleles	Location	Variant Type	MAF (ESA)	CADD Score	RegulomeDB Scores	Final Score	Functional Significance
*CACNA1A*	rs16023	T>A,C	3′ UTR	Post-transcriptional regulatory variant	0.0468	12.3	1f	0.847	Reports related to epilepsy and developmental disorders
*CACNA1A*	rs7249246	T>A,G	Intron	Possible splicing regulatory variant	0.0819	9.8	2b	0.693	GWAS reports related to developmental delay
*CACNA1A*	rs2270655	G>C	Exon	Missense variant	0.0005	15.7	3a	0.874	Related to infantile spasms and epilepsy
*CACNA1C*	rs1006737	G>A	Intron	Regulatory region (enhancer)	0	13.2	1f	0.854	Many reports on autism, epilepsy, and neuropsychiatric disorders
*CACNA1C*	rs4765905	G>A,C	Intron	Regulatory region	0	10.5	2b	0.776	Related to neurological and psychiatric disorders, depression
*CACNA1C*	rs2007044	A>G	Exon	Synonymous variant	0	11	3a	0.851	Related to ASD and developmental disorders
*CACNA1H*	rs2753326	A>G	Intron	Potential enhancer	0	8.7	2b	0.917	Related to absence epilepsy
*CACNA1H*	rs2753325	A>C,G	Intron	Regulatory region	0	9.1	3a	0.841	GWAS reports related to epilepsy
*CACNA1H*	rs2235631	C>A,T	Exon	Missense variant	0	14.5	2b	0.871	Related to pediatric epilepsy

**Table 2:** Demographic and clinical characteristics of pediatric patients with developmental delay/intellectual disability, stratified by epilepsy comorbidity.

Characteristics	Total (*n* = 141) (%)	DD/ID with Epilepsy (*n* = 45) (%)	DD/ID Without Epilepsy (*n* = 96) (%)	*p*-Value
Mean age ± SD (years)	7.36 ± 5.00	10.89 ± 5.26	5.70 ± 3.92	<0.001
Age at epilepsy diagnosis	-	5.50 ± 5.12	-	-
Male (%)	100 (71%)	27 (58%)	69 (72%)	0.868
Severity of DD/ID				0.171
mild	70 (50%)	26 (58%)	44 (46%)	0.253
moderate	45 (32%)	10 (22%)	35 (37%)	0.134
severe/profound	26 (19%)	9 (20%)	17 (18%)	0.925
Motor delay	21 (15%)	4 (9%)	17 (18%)	0.263
Language delay	105 (75%)	37 (82%)	54 (56%)	0.003
Autism spectrum disorders	43 (30%)	15 (33%)	28 (29%)	0.760
Sleep disturbances	15 (%)	4 (%)	11 (%)	0.866
Family history				0.993
DD/ID	22 (16%)	7 (16%)	15 (16%)	1.000
Epilepsy	10 (7%)	8 (18%)	2 (2%)	0.002

**Table 3:** Allele frequency comparison between pediatric patients with DD/ID and the general population.

SNP ID	Gene	Ref (A)/Alt (B)	A (*n*)	B (*n*)	A Frequency	B Frequency	B Frequency * (Controls)	OR (95% CI)	*p*-Value
*CACNA1A*	rs16023	T/A,C	256	26	0.907	0.092	0.0027	37.51 (21.59–65.19)	<0.001
*CACNA1A*	rs7249246	T/A,G	259	23	0.918	0.081	0.0082	10.74 (6.66–17.33)	<0.001
*CACNA1A*	rs2270655	G/C	281	1	0.996	0.003	0.0027	1.31 (0.18–9.71)	0.5415
*CACNA1C*	rs1006737	G/A	282	0	1.0	0.0	0.082	– ^†^	-
*CACNA1C*	rs4765905	G/A,C	282	2	0.992	0.007	0.123	0.03 (0.00–0.18)	<0.001
*CACNA1C*	rs2007044	A/G	282	0	1	0.0	0.191	– ^†^	-
*CACNA1H*	rs2753326	A/G	279	3	0.989	0.010	0.0164	0.64 (0.20–2.03)	0.632
*CACNA1H*	rs2753325	A/C,G	278	4	0.985	0.014	0.0191	0.74 (0.27–2.00)	0.822
*CACNA1H*	rs2235631	C/A,T	278	2	0.992	0.007	0.0109	0.65 (0.16–2.66)	0.771

* B allele frequencies in the control group were obtained from the Genome Aggregation Database (gnomAD, East Asian), and were used as reference values for allele frequency comparison under the assumption of Hardy–Weinberg equilibrium. ^†^ OR values approaching zero or not estimable due to absence of the B allele in the patient group are indicated by “–”.

**Table 4:** Genotype of the 9 SNPs in DD/ID pediatric patients with epilepsy (*n* = 45) and without epilepsy (*n* = 96).

Gene	SNPs	Genotype							*p*-Value
		Alleles	With Epilepsy (*n* = 45)			Without Epilepsy (*n* = 96)			
		A/B	AA	AB	BB	AA	AB	BB	
*CACNA1A*	rs16023	T/A,C	33	7	5	80	15	1	0.0215
*CACNA1A*	rs7249246	T/A,G	41	2	2	88	3	5	0.9105
*CACNA1A*	rs2270655	G/C	45	0	0	95	0	1	1.0
*CACNA1C*	rs1006737	G/A	45	0	0	96	0	0	1.0
*CACNA1C*	rs4765905	G/A,C	45	0	0	95	1	0	1.0
*CACNA1C*	rs2007044	A/G	44	0	0	96	0	0	1.0
*CACNA1H*	rs2753326	A/G	44	1	0	94	1	1	0.6282
*CACNA1H*	rs2753325	A/C,G	44	1	0	94	1	1	0.9805
*CACNA1H*	rs2235631	C/A,T	45	0	0	95	1	0	1.0

## Data Availability

The data are not publicly available due to privacy or ethical restrictions.

## Supplementary Materials

The following supporting information can be downloaded at: https://www.mdpi.com/article/10.3390/genes16070767/s1, Table S1: Allele frequency comparison between pediatric patients with DD/ID and general population.
