# Supplementary material for: Polymorphisms in CACNA1A, CACNA1C, and CACNA1H Genes in Korean Pediatric Patients with Developmental Delay and Intellectual Disability: A Focus on Epilepsy Comorbidity
Source: Genes (Basel). 2025 Jun 29;16(7):767. doi: 10.3390/genes16070767 (PMC12294729; doi:10.3390/genes16070767)
Supplement: Supplementary file 1 [file genes-16-00767-s001.zip › genes-3727458-supplementary.pdf]

Table 1. Allele Frequency Comparison Between Pediatric Patients with DD/ID and General Population

Gene	SNP ID	Ref (A) / Alt (B)	A (n)	B (n)	A frequency	B frequency	B frequency *(Controls)	OR (95% CI)	p-value
CACNA1A	rs16023	T/A,C	256	26	0.907	0.092	0.0468	2.07 (1.37-3.13)	0.0016
CACNA1A	rs7249246	T/A,G	259	23	0.918	0.081	0.0819	1.00 (0.62-1.53)	1.0000
CACNA1A	rs2270655	G/C	281	1	0.996	0.003	0.0005	7.11 (0.83-61.09)	0.1537
CACNA1C	rs1006737	G/A	282	0	1.0	0.0	0.0	-	-
CACNA1C	rs4765905	G/A,C	280	2	0.992	0.010	0.002	3.56 (0.83-15.32)	0.120
CACNA1C	rs2007044	A/G	282	0	1.0	0.0	0.0	-	-
CACNA1H	rs2753326	A/G	279	3	0.989	0.010	0.0087	1.23 (0.39-3.90)	0.7385
CACNA1H	rs2753325	A/C,G	278	4	0.985	0.014	0.0091	1.57 (0.57-4.29)	0.3339
CACNA1H	rs2235631	C/A,T	278	2	0.992	0.007	0.0145	0.49 (0.12-1.98)	0.4435

* B allele frequencies in the control group were obtained from the Korean Reference Genome Database (KRGDB), and were used as reference values for allele frequency comparison under the assumption of Hardy-Weinberg equilibrium. †OR values approaching zero or not estimable due to absence of the B allele in the patient group are indicated by "–".
